# Reliability of the Overnight Dexamethasone Suppression Test in Patients Undergoing Bariatric Surgery

**DOI:** 10.1007/s11695-026-08755-6

**Published:** 2026-05-23

**Authors:** Mehmet Umut Capar, Emre Durcan, Serdar Sahin, Pinar Kadioglu, Mustafa Sait Gonen, Volkan Demirhan Yumuk, Taner Damci, Halit Eren Taskin, Dildar Konukoglu, Candas Ercetin, Ali Tunc, Alperen Ibrahim Sayar, Hande Mefkure Ozkaya

**Affiliations:** 1 Division of Endocrinology and Metabolic Diseases, Department of Internal Medicine, Cerrahpasa Faculty of Medicine, Istanbul University-Cerrahpasa, Istanbul, Turkey; 2 Department of General Surgery, Cerrahpasa Faculty of Medicine, Istanbul University-Cerrahpasa, Istanbul, Turkey; 3 Department of Medical Biochemistry, Cerrahpasa Faculty of Medicine, Istanbul University-Cerrahpasa, Istanbul, Turkey; 4 Department of General Surgery, Bagcilar Training and Research Hospital, University of Health Sciences, Istanbul, Turkey

**Keywords:** Obesity, bariatric surgery, dexamethasone suppression test, cortisol, sleeve gastrectomy, Roux-en-Y gastric bypass

## Abstract

**Introduction:**

Bariatric surgery is the most effective treatment for severe cases in obesity. However, the altered gastrointestinal anatomy after bariatric surgery can affect drug absorption, potentially leading to false-positive results in diagnostic tests. This study aims to evaluate the reliability of dexamethasone suppression test (DST) in post-bariatric surgery patients and explore how different bariatric procedures influence DST outcomes.

**Methods:**

In this single-center, cross-sectional study conducted at the Obesity Center in tertiary university hospital. Patients who had undergone bariatric surgery, completed at least 9–12 months of postoperative recovery, and had a pre-operative DST were included the study. Sociodemographic and clinical data were obtained. All patients underwent an overnight DST and laboratory tests.

**Results:**

Sixty-eight patients included study: 47 sleeve gastrectomy (SG), 21 Roux-en-Y gastric bypass (RYGB). Baseline weight and BMI were similar (*p* = 0.68, *p* = 0.873, respectively). At last follow-up, RYGB patients had lower weight compared to SG (*p* = 0.049). Postoperative DST levels showed no significant change in SG (*p* = 0.055), but increased significantly in RYGB (0.72 vs. 0.84; *p* = 0.028). Although postoperative DST values were higher in RYGB, the difference between groups was not significant (*p* = 0.196). A moderate positive correlation was found between pre- and postoperative DST values (*r* = 0.464, *p* < 0.001), with no association between DST and sex, surgery type, BMI, or weight loss.

**Conclusions:**

Despite potential alterations in absorption after bariatric surgery, the DST appeared to remain reliable and adequately suppressed cortisol in the majority of patients. Thus, our findings suggest that DST may be considered a safe and valid screening tool for Cushing’s syndrome in patients undergoing bariatric surgery.

## Key Points

Suppression of DST was achieved in 94.2% of patients undergoing bariatric surgery.

Although postoperative DST levels increased significantly in the RYGB group, almost all remained suppressed (0.72 vs. 0.84; *p* = 0.028).

Our data suggest that overnight DST may be used safely in patients undergoing sleeve gastrectomy and RYGB.

## Introduction

Obesity is a chronic, multifactorial and recurrent disease driven by biological, behavioral, and environmental factors, and its global prevalence continues to rise [1–3]. Treatment options include lifestyle interventions (diet, exercise and behavioral therapy), pharmacotherapy, and bariatric surgery, with the latter providing the most durable weight loss, substantial improvement in associated medical problems, enhanced quality of life, and reduced mortality in patients with severe obesity [4–8]. After surgery, the focus shifts towards long-term weight maintenance and sustained cardiometabolic benefits [9–18]. Globally, sleeve gastrectomy (SG) is the most commonly performed primary bariatric procedure, followed by Roux-en-Y gastric bypass (RYGB) [19].

Anatomical alterations and weight loss after bariatric surgery can influence drug absorption, distribution, and dosing. Drug absorption depends on physicochemical properties such as solubility, lipophilicity, and molecular size, while post-surgical changes in gastric pH, emptying time, mucosal surface, and intestinal length further modify drug disposition [20–22]. Although several studies have investigated drug absorption after bariatric procedures, the complex and multifactorial physiological changes make it difficult to establish standardized dosage recommendations, even for drugs metabolized predominantly by the same enzyme.

In patients undergoing bariatric surgery, Cushing’s syndrome (CS) can often be overlooked as a secondary etiology, and delays in diagnosis can lead to irreversible complications. Recognizing CS before or after bariatric surgery is critical for the overall health of patients. In particular, the overlap of symptoms with obesity-related conditions can complicate the diagnosis of CS [23, 24]. The literature has shown delays of up to several years in the diagnosis of CS in patients undergoing bariatric surgery [24–26]. Postoperatively, these patients may experience complications such as thromboembolism, osteoporosis, and malnutrition, significantly increasing morbidity and mortality [23–25]. Therefore, screening for CS before bariatric surgery is crucial to prevent complications that may develop after surgery [23].

In clinical practice, endocrine causes of recurrent weight gain or suboptimal clinical response after bariatric surgery often warrant re-evaluation. The overnight dexamethasone suppression test (DST) is frequently used in these patients to assess for CS as a secondary etiology. After bariatric surgery, dexamethasone tablets may not dissolve adequately for sufficient absorption due to altered gastrointestinal anatomy. This can result in false-positive results in DSTs for CS, as both pharmacokinetic and pharmacodynamic processes are affected. In the present study, we aimed to evaluate the reliability of DST in the post-bariatric surgery population and the effect of different bariatric surgical procedures on DST.

## Materials and Methods

### Participants and Procedure

**Fig. 1:**
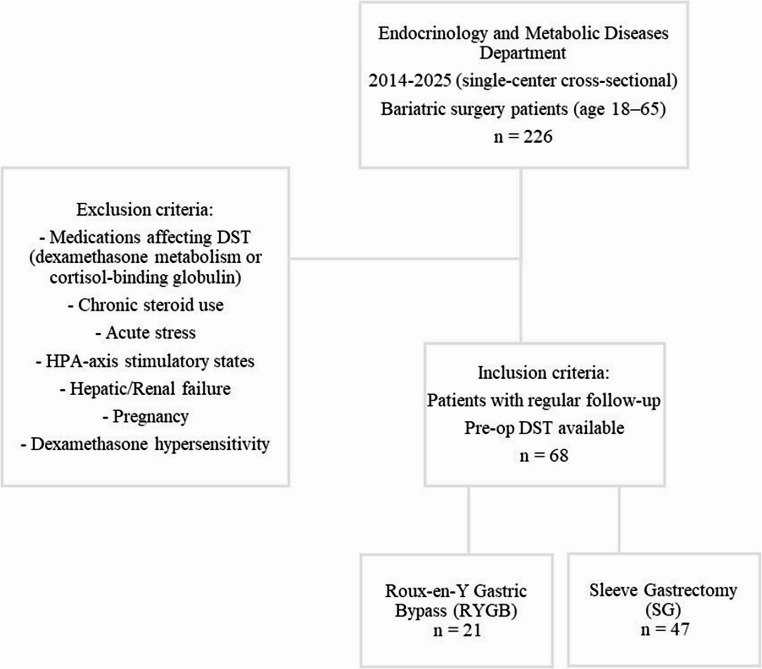
Flow diagram of patient admission to the study

In this single-center cross-sectional study, patients followed at EASO Collaborating Center for Obesity Management within the Division of Endocrinology and Metabolic Diseases, Istanbul University-Cerrahpasa between 2014 and 2025 were included (Fig. 1). Individuals who presented to our center for bariatric surgery were evaluated for secondary causes of obesity. Those without any identifiable secondary cause were assessed by a multidisciplinary team consisting of an endocrinologist, bariatric surgeon, psychologist/psychiatrist, dietitian, and exercise specialist, and were subsequently referred for bariatric surgery. An overnight DST was administered to all patients prior to surgery.

The inclusion criteria were: (i) having undergone bariatric surgery, and (ii) being between 18 and 65 years of age. Exclusion criteria included the use of medications known to interfere with DST results by altering dexamethasone metabolism or cortisol-binding globulin levels (e.g., phenobarbital, phenytoin, carbamazepine, primidone, rifampin, ethosuximide, pioglitazone, aprepitant, itraconazole, ritonavir, fluoxetine, diltiazem, cimetidine, oral contraceptives, and mitotane); chronic corticosteroid therapy for other indications; acute stress states such as severe illness, infection, or postoperative recovery; conditions stimulating the hypothalamic–pituitary–adrenal axis; hepatic or renal failure; pregnancy; and known hypersensitivity to dexamethasone.

An a priori sample size calculation was performed using G*Power software based on pre- and postoperative DST cortisol values reported by Casteràs et al. (0.82 ± 0.29 vs. 0.94 ± 0.31 µg/dL), corresponding to an effect size of 0.40. Using a two-sided alpha of 0.05 and a statistical power of 0.85 for paired comparisons, the required sample size was estimated to be 59 participants [27].

Ethical approval was obtained from the Istanbul University-Cerrahpasa Medical Research Ethics Committee (E-83045809-604.01-910159). Signed informed consent was obtained from all study participants.

### Data Collection

Sociodemographic data, associated medical conditions, current medications (including duration and dosage), dates of bariatric surgery, preoperative laboratory results, and preoperative height, weight, and BMI were retrospectively obtained from archived medical records. The age at diagnosis of obesity (defined as the date when BMI ≥ 30 kg/m²), history of medical treatment for obesity (e.g., orlistat, exenatide, liraglutide, semaglutide), duration of these treatments, prior gastric balloon therapy, and the type of bariatric procedure performed were also recorded.

During routine follow-up visits conducted at least 9–12 months postoperatively (when gastric adaptation was considered complete), patients underwent reassessment of height, weight, body mass index (BMI), weight loss, and laboratory parameters. These included complete blood count, renal function tests, aspartate aminotransferase (AST), alanine aminotransferase (ALT), fasting plasma glucose (FPG), insulin, glycated hemoglobin (HbA1c), homeostatic model assessment of insulin resistance (HOMA-IR), lipid profile, thyroid function tests, adrenocorticotropic hormone (ACTH), cortisol, serum iron, total iron-binding capacity (TIBC), ferritin, vitamin B12, and vitamin D.

### Measurements

Patients with a BMI *≥* 30 kg/m² were diagnosed as having obesity and underwent further evaluation [28]. Insulin resistance was assessed using the HOMA-IR. All patients received a 1 mg oral dexamethasone tablet at 23:00, and blood samples were collected between 08:00 and 09:00 the following morning for plasma cortisol measurement. Adequate dexamethasone absorption was evaluated based on the presence of cortisol suppression. Cushing’s syndrome was excluded in patients with DST results < 1.8 µg/dL in the absence of suggestive clinical findings [29].

While the samples were being studied, the blood sample in the serum tube was centrifuged, and the serum was separated. Another tube containing tripotassium–ethylenediaminetetraacetic acid (K₃-EDTA) was used for ACTH, HbA1c, and complete blood count (CBC) analyses. Serum vitamin B12, vitamin D, insulin, thyroid function tests, ferritin, and cortisol levels were measured using Elecsys Cortisol Generation II electrochemiluminescence competitive immunoassay kits (Cobas e801, Roche Diagnostics GmbH, Mannheim, Germany). Serum AST, urea, creatinine, lipid profile, serum iron, TIBC, and FPG were analyzed using colorimetric methods (Cobas c701, Roche Diagnostics GmbH, Mannheim, Germany). Complete blood count analyses were performed on the Sysmex XN-series analyzer (Sysmex Europe GmbH, Germany). Glycated hemoglobin (HbA1c) was measured using an automated hemoglobin analyzer (Lifotronic H9, Lifotronic Technology Co., Ltd., China).

### Statistical Analysis

Statistical analyses were conducted using the Statistical Package for the Social Sciences (SPSS, version 25.0, USA). The distribution of variables was assessed with the Kolmogorov–Smirnov test. Continuous variables with a normal distribution are presented as mean ± standard deviation (SD), while non-normally distributed continuous variables are expressed as median [interquartile range, IQR]. Categorical variables are reported as numbers and percentages. For comparisons between two groups, the independent samples t-test was used for normally distributed quantitative variables, and the Mann–Whitney U test was used for non-normally distributed quantitative variables. The Chi-square test was used for categorical variables; when Chi-square assumptions were not met, Fisher’s exact test was used. To compare the relationship between pre- and post-operative DST values, the difference between the two (ΔDST) and the percentage change (%DST) were used. Positive changes between DSTs indicate an increase, while negative changes indicate a decrease. ΔDST and %DST were calculated using the following formulas. ΔDST = Post-op DST - Pre-op DST and %DST = (Post-op DST - Pre-op DST) / Pre-op DST*100. To address complementary analytical objectives, both correlation analyses and threshold-based subgroup comparisons were performed. Spearman’s rank correlation coefficient was used to examine associations between DST values and continuous variables, including time since surgery. Correlation analysis assessed linear relationships across the entire follow-up period, whereas threshold-based subgroup analyses were used to identify potential nonlinear or time-dependent effects that may not be detected by correlation testing. A p-value < 0.05 was considered statistically significant, with a confidence level of 95%.

## Results

### Patients’ Characteristics

A total of 68 patients underwent bariatric surgery: 47 had SG and 21 had RYGB. In the SG group, 35 patients (74.5%) were female, while 14 patients (66.7%) were female in the RYGB group. The mean age was 43.9 ± 12.3 years in the SG group and 50.5 ± 10 years in the RYGB group. In the SG group, 13 patients (27.7%) had diabetes mellitus, 4 (8.5%) had hyperlipidemia, and 16 (34.0%) had hypertension. In the RYGB group, 6 patients (28.6%) had diabetes mellitus, 5 (23.8%) had hyperlipidemia, and 2 (9.5%) had hypertension. The two groups were comparable in terms of sex distribution and associated medical conditions (*p* > 0.05 for all); however, a significant difference was observed with respect to age (*p* = 0.039) (Table 1).

**Table 1:** Characteristics of patients undergoing bariatric surgery

	Sleeve gastrectomy (*n* = 47)		RYGB (*n* = 21)	*p* value
	*n* (%), mean ± SD or median [IQR]			
Sex, female		35 (74.5)	14 (66.7)	0.711
Age, *years*		43.89 ± 12.32	50.52 ± 10.04	*0.039*
Age of diagnosis, years		27.12 ± 14.80	33.85 ± 15.48	0.084
Single/Married		14/33 (29.8/70.2)	5/16 (23.8/76.2)	0.830
Education level, > 12 years		14 (29.8)	4 (19)	0.728
Diabetes Mellitus		13 (27.7)	6 (28.6)	1.000
Hyperlipidemia		4 (8.5)	5 (23.8)	0.122
Hypertension		16 (34)	2 (9.5)	0.069
Coronary Artery Disease		0 (0)	1 (4.8)	0.309
Peripheral Arterial Disease		1 (2.1)	0 (0)	1.000
Hypothyroidism		9 (19.1)	5 (23.8)	0.749
Smoker		21 (44.7)	12 (57.1)	0.492
Alcohol use		8 (17)	5 (23.8)	0.520
Substance use		0 (0)	0 (0)	1.000
Psychiatric disorders		6 (12.8)	3 (14.3)	1.000
Post-operative follow-up time, month		86 [36.5–105.0]	51 [18.5–82.0]	*0.030*
Pre-operative weight, kg		128 [115–156]	127 [116.5-146.5]	0.681
Pre-operative BMI, kg/m²		44.8 [41.6–52.7]	45.4 [40.0-55.7]	0.873
Last follow-up weight, kg		80 [70–94]	75 [69–80]	*0.049*
Last follow-up BMI, kg/m²		28.3 [25.2–35.1]	27.8 [24.2–31.3]	0.198
Maximum weight lost, kg		46.2 [37.0–55.0]	53.0 [38.0-68.5]	0.282
Pre-operative medical therapy No Orlistat Exenatide Liraglutide Orlistat + Exenatide Orlistat + Exenatide + Liraglutide		34 (72.3) 5 (10.6) 1 (2.1) 6 (12.8) 0 (0) 1 (2.1)	13 (61.9) 4 (19) 0 (0) 1 (4.8) 2 (9.5) 1 (4.8)	0.208
Duration of medical therapy, month		2.5 [1.25–9.75]	4.5 [2.00-13.75]	0.385
Intragastric balloon		1 (2.1)	1 (4.8)	0.525

*SD* standard deviation *IQR* interquartile range *BMI* body mass index, RYGB roux-en-Y Gastrik Bypass

The median preoperative weight and BMI were 128 [115–156] kg and 44.8 [41.60–52.7] kg/m² in the SG group, and 127 [116.5–146.5] kg and 45.4 [40.0–55.7] kg/m² in the RYGB group. There were no significant differences in preoperative weight or BMI between the groups (*p* = 0.68, *p* = 0.873, respectively). At the last follow-up visit, the median weight and BMI were 80 [70–94] kg and 28.3 [25.2–35.1] kg/m² in the SG group, and 75 [69–80] kg and 27.8 [24.2–31.3] kg/m² in the RYGB group. Postoperative body weight differed significantly between the two groups (*p* = 0.049); however, maximum weight loss was comparable (*p* = 0.282) (Table 1).

There were no significant differences in preoperative laboratory findings (including anemia markers, nutritional parameters, liver function tests, lipid profile, glucose, and HbA1c) or DST results between the SG and RYGB groups (Table 2).

Patients’ laboratory findings are summarized in Table 2.

**Table 2:** Comparison of preoperative laboratory values of patients undergoing sleeve gastrectomy or RYGB

	Sleeve gastrectomy (*n* = 47)	RYGB (*n* = 21)	*p* value
	*n* (%) or median [IQR]		
Overnight DST (µg/dL)	0.69 [0.49–0.86]	0.72 [0.54–0.92]	0.610
Hemoglobin (g/dl)	13.30 [12.25–14.37]	13.45 [11.25–14.67]	0.585
AST (IU/L)	19 [16–25]	15 [14–21]	0.120
ALT (IU/L)	21 [15–31]	20 [12–30]	0.330
FPG (mg/dL)	89 [81–107]	96.00 [82.25-121.25]	0.381
HbA1c (%)	6.0 [5.4–6.7]	5.8 [5.5–8.1]	0.842
Insulin (µU/ml)	16.53 [9.38–29.51]	13.70 [6.15–21.45]	0.314
HOMA-IR	3.69 [2.55–9.12]	3.48 [2.16–5.87]	0.332
Total cholesterol (mg/dL)	183 [167–215]	190.50 [159.25-236.25]	0.599
LDL (mg/dL)	113 [96–147]	129.90 [93.25–165.00]	0.470
HDL (mg/dL)	42 [37–55]	48 [41–59]	0.292
Triglyceride (mg/dL)	140 [114–177]	140 [90–172]	0.447
Fe (µg/dL)	61 [49–79]	63 [45–67]	0.579
TIBC (µg/dL)	376 [357–398]	365 [307–390]	0.266
Ferritin (ug/L)	48.9 [19.2-114.7]	38.7 [22.7–63.5]	0.444
Vitamin B12 (pg/ml)	332 [259–414]	301 [235–401]	0.407
Vitamin D (µg/L)	16.2 [10.7–22.5]	18.1 [12.8–25.7]	0.437

*IQR* interquartile range *DST* dexamethasone suppression test *FPG* fasting plasma glucose, *HOMA-IR* homeostatic model assessment for ınsulin resistance, *LDL* low-density lipoprotein, *HDL* high-density lipoprotein, *Fe* iron, *TIBC* total iron binding capacity

## Effect of time Since Surgery

In the post-operative period, the median ΔDST was 0.082 [(-0.054) − 0.255] and %DST was 11.64 [(-7.22) − 42.25] in the entire group. When we divided the patients into two groups during the period when we applied DST, taking the time elapsed since bariatric surgery as the 12-month cut-off, there was no difference between the two groups in terms of ΔDST and %DST results (*p* = 0.675 and *p* = 0.313, respectively). Using a 24-month threshold, DST was performed in 16 patients within the first 24 months after surgery and in 52 patients beyond 24 months. In the < 24 months group, median ΔDST was 0.135 [0.067–0.403] and %DST was 26.56 [10.60–66.45]. In contrast, in the > 24 months group, median ΔDST was 0.049 [(-0.095) − 0.208] and %DST was 6.14 [(-17.55) − 27.77]. Both ΔDST and %DST were significantly higher when DST was performed within the first 24 months after surgery (*p* = 0.046 and *p* = 0.018, respectively).

## Comparison of Pre-Operative and last Follow-up Parameters Separately Within Groups

In the SG group, significant reductions in weight, BMI, AST, ALT, FPG, HbA1c, insulin, HOMA-IR, and triglyceride levels were observed from baseline to the last follow-up, consistent with the overall cohort. However, no significant change was observed in the DST (*p* = 0.055) (Table 3).

**Table 3:** Pre-operative and last follow-up comparison of patients who underwent sleeve gastrectomy

	Pre-operative	Last follow-up	*p* value
	*n* (%), mean ± SD or median [IQR]		
Overnight DST (µg/dL)	0.69 [0.49–0.86]	0.66 [0.49–1.07]	0.055
Weight, *kg*	128 [115–156]	80 [70–94]	*< 0.001*
BMI, *kg/m²*	44.8 [41.6–52.7]	28.3 [25.2–35.1]	*< 0.001*
Hemoglobin (g/dl)	13.30 [12.25–14.37]	12.95 [11.92–14.02]	0.257
AST (IU/L)	19 [16–25]	17 [14–21]	*0.002*
ALT (IU/L)	21 [15–31]	15 [10–19]	***< *** *0.001*
FPG (mg/dL)	89 [81–107]	85 [80–95]	*0.024*
HbA1c (%)	6.0 [5.4–6.7]	5.5 [5.0-5.8]	***< *** *0.001*
Insulin (µU/ml)	16.53 [9.38–29.51]	9.44 [6.50-12.35]	*0.006*
HOMA-IR	3.69 [2.55–9.12]	1.90 [1.40–2.84]	*0.002*
Total cholesterol (mg/dL)	183 [167–215]	187 [174–228]	0.507
LDL (mg/dL)	113 [96–147]	119 [104–150]	0.682
HDL (mg/dL)	42 [37–55]	57 [49–68]	***< *** *0.001*
Triglyceride (mg/dL)	140 [114–177]	104 [80–130]	***< *** *0.001*
Fe (µg/dL)	61 [49–79]	74 [50–97]	0.553
TIBC (µg/dL)	376 [357–398]	363 [278–394]	0.354
Ferritin (ug/L)	48.9 [19.2-114.7]	23.1 [12.9–76.0]	*0.049*
Vitamin B12 (pg/ml)	332 [259–414]	313 [265–453]	0.829
Vitamin D (µg/L)	16.2 [10.7–22.5]	18.7 [12.6–28.3]	0.638

*SD* standard deviation, *IQR* interquartile range, *DST* dexamethasone suppression test, *FPG* fasting plasma glucose, *HOMA-IR* homeostatic model assessment for ınsulin resistance, *LDL* low-density lipoprotein, *HDL* high-density lipoprotein, *Fe* iron, *TIBC* total iron binding capacity

In the RYGB group, weight, BMI, AST, FPG, HbA1c, insulin, and HOMA-IR levels significantly decreased from baseline to the last follow-up. In contrast, DST levels showed a significant increase (0.72 vs. 0.84; *p* = 0.028) (Table 4).

**Table 4:** Pre-operative and last follow-up comparison of patients who underwent RYGB

	Pre-operative	Last follow-up	*p* value
	*n* (%), mean ± SD or median [IQR]		
Overnight DST (µg/dL)	0.72 [0.54–0.92]	0.84 [0.67–0.98]	*0.028*
Weight, *kg*	127 [116.5-146.5]	75 [69–80]	***< *** *0.001*
BMI, *kg/m²*	45.4 [40.0-55.7]	27.8 [24.2–31.3]	***< *** *0.001*
Hemoglobin (g/dl)	13.45 [11.25–14.67]	12.90 [12.00-13.40]	0.444
AST (IU/L)	15 [14–21]	20 [15–27]	*0.036*
ALT (IU/L)	20 [12–30]	18 [13–30]	0.387
FPG (mg/dL)	96.00 [82.25-121.25]	89 [80–99]	*0.025*
HbA1c (%)	5.8 [5.5–8.1]	5.6 [5.2–6.5]	*0.042*
Insulin (µU/ml)	13.70 [6.15–21.45]	6.71 [5.11–9.02]	*0.050*
HOMA-IR	3.48 [2.16–5.87]	1.37 [1.04–2.10]	*0.017*
Total cholesterol (mg/dL)	190.50 [159.25-236.25]	174 [153–205]	0.478
LDL (mg/dL)	129.90 [93.25–165.00]	99 [85–133]	0.193
HDL (mg/dL)	48 [41–59]	54 [48–64]	0.327
Triglyceride (mg/dL)	140 [90–172]	91 [63–142]	0.136
Fe (µg/dL)	63 [45–67]	59 [40–88]	0.205
TIBC (µg/dL)	365 [307–390]	381 [334–429]	0.679
Ferritin (ug/L)	38.7 [22.7–63.5]	36.5 [10.3-107.2]	0.156
Vitamin B12 (pg/ml)	301 [235–401]	354 [226–449]	0.145
Vitamin D (µg/L)	18.1 [12.8–25.7]	24.6 [14.3–29.7]	0.339

*SD* standard deviation, *IQR* interquartile range, *DST* dexamethasone suppression test, *FPG* fasting plasma glucose, *HOMA-IR* homeostatic model assessment for ınsulin resistance, *LDL* low-density lipoprotein, *HDL* high-density lipoprotein, *Fe* iron, *TIBC* total iron binding capacity

### Comparison of DST at last Follow-up Between Groups

At the postoperative last follow-up, four patients exhibited non-suppressed overnight DST results. Three of them were in the SG group and one was in the RYGB group (Table 5). The median DST value was 0.66 [0.49–1.07] in the SG group and 0.84 [0.67–0.98] in the RYGB group. Although DST values were tended to be higher in the RYGB group, the difference did not reach statistical significance (*p* = 0.196).

**Table 5:** Multiple linear regression analysis evaluating factors associated with postoperative DST

Table 5. Characteristics of 4 patients with non-suppressed DST after bariatric surgery					
Patient	1	2	3	4	
Group	SG	SG	SG	RYGB	
Gender	Male	Female	Female	Male	
Age, years	64	51	60	65	
Post-op follow-up time, month	122	99	93	84	
Diabetes mellitus	Yes	Yes	Yes	Yes	
Hyperlipidemia	No	No	No	Yes	
Hypertension	Yes	Yes	Yes	No	
Pre-op BMI, kg/m²	48.1	67.2	54	35.9	
Pre-op overnight DST (µg/dL)	2.00	0.87	3.10	2.57	
Last follow-up					
Cushing’s stigmata	No	No	No	No	
BMI, kg/m²	35.1	33.2	36.7	27.6	
Overnight DST (µg/dL)	1.99	2.07	3.30	3.71	
ACTH (pg/mL)	10.1	11.2	12.30	28.0	
Cortisol (µg /dL)	14.6	14.4	13.8	8.5	
24-h UFC (mg/day)	37	26	39	43	
LNSC (µg/dL)	0.13	0.18	0.14	0.21	
Albumine (gr/dL)	4.47	4.75	4.56	4.44	
Ferritin (ug/L)	105.0	19.3	10.7	39.0	
Vitamin B12 (pg/ml)	220	756	2000	256	
Vitamin D (µg/L)	9.95	8.12	20.90	14.60	

*DST* dexamethasone suppression test, *SG* sleeve gastrectomy, *RYGB* roux-en-Y gastric bypass, *BMI* body mass index, *ACTH* adrenocorticotropic hormone, *UFC* urinary free cortisol, *LNSC* late-night salivary cortisol

Of the four patients with non-suppressed postoperative DST results, three also demonstrated non-suppressed DST values preoperatively, indicating persistent non-suppression rather than a post-operative change. Only one patient showed conversion from a suppressed preoperative DST to a non-suppressed postoperative DST.

**Fig. 2:**
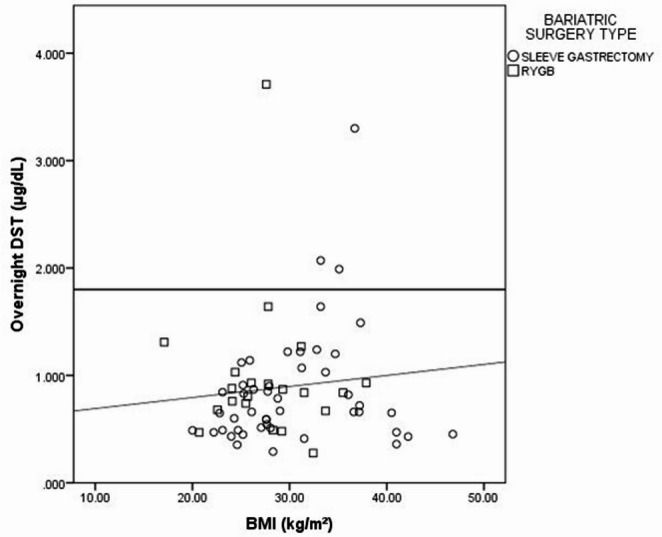
Correlation analysis between overnight DST and BMI at last follow-up (Circles represent patients who underwent sleeve gastrectomy, and squares represent patients who underwent Roux-en-Y gastric bypass. The horizontal line indicates the cortisol cut-off value of 1.8 µg/dL for adequate suppression. Linear regression analysis demonstrated no significant association between BMI and overnight DST levels (R² = 0.011, *p* > 0.05))

A moderate positive correlation was observed between preoperative and last follow-up DST levels (*r* = 0.464, *p* < 0.001). No significant correlations were identified between overnight DST levels (preoperative or last follow-up) and time since surgery, preoperative BMI, last follow-up BMI, weight loss, ferritin, vitamin B12 and D-vitamin levels (Fig. 2).

Finally, to address the baseline differences between the surgical groups, a multiple linear regression analysis was performed to evaluate the factors associated with the change in DST (ΔDST). After adjusting for age, postoperative follow-up time, and last follow-up weight, the type of bariatric surgery (RYGB vs. SG) was not found to be an independent predictor of postoperative ΔDST (B = 0.013, 95% CI: -0.236 to 0.262, *p* = 0.917) (Table 6).

**Table 6:** Multiple linear regression analysis evaluating factorsassociated with postoperative ΔDST

Δ			
Variables	Unstandardized B	95% Confidence Interval	*p* value
Surgery type (RYGB vs. SG)	0.013	-0.236–0.262	0.917
Age, *years*	0.002	-0.007–0.012	0.611
Post-operative follow-up time, *month*	-0.001	-0.003–0.002	0.613
Last follow-up weight, *kg*	0.001	-0.005–0.007	0.683

*ΔDST* last follow-up DST – pre-operative DST, *RYGB* roux-en-Y Gastrik Bypass, *SG* sleeve gastrectomy

## Discussion

In this study, we evaluated the reliability of the DST in bariatric surgery patients who underwent SG or RYGB. Although post-operative DST values were slightly higher in the RYGB group compared to the SG group, there was no significant difference between the groups. In addition, our findings show that the standard overnight DST appeared to provide reliable results in both surgical groups, with adequate cortisol suppression achieved in nearly all patients.

Metabolic improvements are generally expected after bariatric surgery [30, 31]. Our study confirmed these benefits in both the overall cohort and individual surgical subgroups. Significant reductions were observed in FPG, HbA1c, insulin, and HOMA-IR levels compared with preoperative values. Triglyceride levels also decreased, and HDL levels increased, although the latter did not reach statistical significance. These findings are consistent with previous reports demonstrating the impact of bariatric surgery on improving glycemic control and reducing HbA1c [30–34]. Vitamin deficiencies, particularly vitamin D deficiency, are frequently reported after bariatric procedures due to impaired nutrient absorption. For example, a meta-analysis by Chen et al. including 45 studies found a prevalence of vitamin D deficiency as high as 35.8% [35]. In contrast, our study showed higher postoperative vitamin D levels compared with baseline. This discrepancy is likely attributable to the fact that patients in our cohort were closely monitored and received adequate supplementation, which effectively prevented deficiency. Furthermore, due to the long postoperative follow-up period, gastric adaptation may have been completed by this time, thus reducing absorption problems.

Bariatric surgery is known to modify not only nutrient absorption but also drug bioavailability through reduced gastric surface area, altered gastric emptying, bypass of the proximal intestine, changes in gastric pH, and disruption of CYP450 activity in the duodenum and proximal jejunum [20–22, 36, 37]. Pharmacokinetic effects vary by drug class. Roerig et al. reported significantly lower AUC and peak plasma concentrations of antidepressants after RYGB, whereas Padwal et al. demonstrated increased metformin absorption and bioavailability in a single-dose in post-RYGB patients [38, 39]. Similarly, Rubio et al. found that levothyroxine absorption was preserved but considerably delayed following RYGB [40].

Corticosteroids are lipophilic compounds with similar molecular structures, and their oral bioavailability, including that of dexamethasone, generally ranges from 60% to 100% [37]. Enteral absorption of corticosteroids is believed to be higher in the proximal small intestine and lower in the distal segments [41]. There are also reports on steroid pharmacokinetics following bariatric surgery. Heide et al. investigated cortisol profiles in five patients with primary or secondary adrenal insufficiency before and after different bariatric procedures and found nearly identical results, suggesting preserved steroid absorption [42]. In line with these findings, our study demonstrated adequate suppression with the 1 mg dexamethasone in patients without clinical features of CS, further suggesting that steroid absorption and bioavailability may not be significantly impaired after bariatric surgery in this clinical context.

In our study, no patient was diagnosed with CS. Of these, 64 patients (94.2%) showed suppressed DST levels both preoperatively and postoperatively. Four patients had unsuppressed DST values after bariatric surgery: three after SG and one after RYGB. Two SG patients and one RYGB patient also had unsuppressed preoperative DST results. Only one patient (SG) showed suppression preoperatively but loss of suppression postoperatively. Importantly, three of these patients had non-suppressed DST values already present preoperatively, representing persistent non-suppression rather than a surgery-related change. Only one patient demonstrated postoperative conversion from suppression to non-suppression, which is the most relevant scenario to evaluate post-bariatric DST reliability. None of these patients exhibited clinical features of CS before or after surgery. Further testing, including two 24-hour urinary free cortisol collections, late-night salivary cortisol, and a 2-day 2 mg DST, yielded normal results. These findings were therefore classified as non-suppressed DST results with negative confirmatory testing, rather than evidence of impaired dexamethasone absorption. In the Mannheim Obesity Study, Lammert et al. applied DST to 278 patients with severe obesity and found that inadequate suppression was rare (8.6%), confirmed hypercortisolism was extremely uncommon (< 1%), and the specificity of DST was 92% [43]. Consistent with these findings, our results suggest that the DST may be a safe and effective screening tool for CS in bariatric surgery patients within the study’s scope.

Some studies in the literature have expressed concerns about possible impairments in dexamethasone absorption and DST suppression in post-bariatric surgery patients. Humpert et al. described two bariatric patients in whom suppression was not achieved with 2 mg and 4 mg overnight DSTs, attributing this to impaired corticosteroid absorption [44]. Thomas et al. reported a higher rate of acute rejection in RYGB patients following kidney transplantation, suggesting reduced immunosuppressant bioavailability [45]. Casteràs et al., in a study of 38 individuals (21 patients who underwent bariatric surgery two years ago, 10 patients with severe obesity without bariatric surgery, and 7 healthy controls), found significantly higher morning cortisol levels after DST in the post-bariatric group compared with preoperative values (0.90 vs. 0.70 µg/dL; *p* < 0.01). Four patients (4/21, 19%) in the post-bariatric surgery group exhibited cortisol levels above the diagnostic cut-off (> 1.8 µg/dL) despite the absence of autonomous cortisol secretion, representing non-suppressed DST results. In addition, plasma dexamethasone concentrations were significantly lower in post-bariatric surgery patients compared with both non-operated individuals with obesity and healthy controls (1.90 vs. 3.70 vs. 4.00 ng/dL; *p* < 0.01) [46]. Based on these findings, the authors highlighted potential limitations in DST interpretation after bariatric surgery and emphasized the role of altered dexamethasone pharmacokinetics rather than true hypercortisolism [46]. In our study, when pre-operative and post-operative DST values were compared, while there was no difference in the SG group post-operative DST values were significantly higher in the RYGB group. Our findings are partially similar to those of Casteràs et al. regarding the initial trend toward higher DST values in RYGB; however, our adjusted analysis further clarifies that this trend is likely driven by confounding factors such as age and follow-up duration rather than the procedure itself. On the other hand, our study had a larger sample size and the separate analysis of SG and RYGB groups. Furthermore, when we evaluated the entire cohort in terms of ΔDST and %DST based on 24 months, our data suggests that DST values were higher in the first two years after surgery, and this difference decreased after the second year, possibly due to improved gastric anatomic adaptation. These findings suggest that anatomic adaptation may also be prolonged. Taking into account the false-positive DST results obtained in our and previous studies [46], we recommend thoroughly examining and investigating patients with positive DSTs, especially in the first two years, and repeating the DST after the second year, if possible.

It should be acknowledged that the sleeve gastrectomy and Roux-en-Y gastric bypass groups differed with respect to age, postoperative follow-up duration, and postoperative body weight. These factors may act as potential confounders influencing dexamethasone pharmacokinetics and DST dynamics, independent of surgical procedure. Older age has been associated with subtle alterations in cortisol metabolism [47], while longer follow-up duration and greater postoperative weight loss may reflect different stages of metabolic and anatomical adaptation after surgery.

Consequently, procedure-specific effects cannot be definitively confirmed due to these significant confounding factors. Therefore, the observed increase in postoperative DST values particularly within the RYGB group should be interpreted with caution and not attributed solely to procedure-specific effects. Indeed, our multiple regression model confirmed that surgery type lost its significance when other clinical variables were accounted for. Rather, these findings likely reflect a complex interaction between surgical anatomy, patient characteristics, and time-dependent postoperative changes.

Our study has some limitations. First, pre-operative plasma dexamethasone levels could not be measured. Secondly, post-operative DST measurements taken at different times may have been obtained at different stages of weight loss or metabolic adaptation. On the other hand, we included patients whose DST were obtained at least 9–12 months after the surgery. According to previous prospective studies, metabolic adaptation after bariatric surgery becomes most evident during the rapid weight loss phase at 3 months and is largely complete after 12 months [48]. Additionally, we performed a post-hoc analysis to evaluate the effect of time on DST levels, and our results suggest that 24th month after bariatric surgery is more reliable in assessing gastric adaptation. The study was cross-sectional rather than prospective, and potential temporal changes in glucocorticoid metabolism may have occurred. Furthermore, the significant differences between the SG and RYGB groups regarding age and follow-up duration limit our ability to isolate the independent impact of each surgical procedure on DST outcomes.

This study provides one of the largest real-world datasets evaluating the diagnostic performance of DST performed after bariatric surgery. Unlike most previous studies, which are small and highly selective, our cross-sectional design reflects routine clinical practice conditions and therefore offers valuable validity. The inclusion of both SG and RYGB patients allows for direct comparisons between different types of surgery; this distinction is rarely analyzed in the existing literature. Although plasma dexamethasone levels were not measured, our study’s detailed characterization of clinical and biochemical parameters including BMI change, micronutrient indices (vitamin D, ferritin, B12, albumin), and medication use allows for an indirect assessment of potential factors influencing DST variability. Importantly, our findings were derived from a national cohort, representing one of the first analyses of post-bariatric endocrine test performance in this population. Given potential ethnic and procedural differences in pharmacokinetics and surgical techniques, this regional contribution fills an important gap in the global evidence base. Careful exclusion of confounding factors further enhances internal validity.

## Conclusion

Although changes in drug and nutrient absorption are expected after bariatric surgery, our findings suggest that DST, an important screening tool for CS may remain a reliable tool and provided adequate suppression in the vast majority of our patients. Therefore, determining the reliability of DST may help reduce the financial burden of unnecessary investigations and minimize the risk of misdiagnosis and inappropriate treatment. The inclusion of both the SG and RYGB cohorts provides new comparative information on how different surgical anatomies may influence DST outcomes, even without measured plasma dexamethasone levels. A trend toward increased postoperative DST values was observed in the RYGB group, suggesting that absorption issues should be considered, but suppression was almost consistently achieved especially 24 months after surgery. These results suggest that contrary to common concerns, DST appears to be safely used in screening for CS in bariatric surgery. 

## Data Availability

The data underlying this article cannot be shared publicly due to ethical reasons and for the privacy of individuals that participated in the study. The data will be shared on reasonable request to the corresponding author.
